# Prevalence of physical inactivity in Iran: a systematic review

**DOI:** 10.15171/jcvtr.2016.20

**Published:** 2016-09-30

**Authors:** Hossein Fakhrzadeh, Shirin Djalalinia, Mojdeh Mirarefin, Tahereh Arefirad, Hamid Asayesh, Saeid Safiri, Elham Samami, Morteza Mansourian, Morteza Shamsizadeh, Mostafa Qorbani

**Affiliations:** ^1^Elderly Health Research Center, Endocrinology and Metabolism Population Sciences Institute, Tehran University of Medical Sciences, Tehran, Iran; ^2^Non-communicable Diseases Research Center, Endocrinology and Metabolism Population Sciences Institute, Tehran University of Medical Sciences, Tehran, Iran; ^3^Development of Research & Technology Center, Deputy of Research and Technology, Ministry of Health and Medical Education, Tehran, Iran; ^4^Chronic Diseases Research Center, Endocrinology and Metabolism Population Sciences Institute, Tehran University of Medical Sciences, Tehran, Iran; ^5^Department of Exercise Physiology, Science and Research Branch, Islamic Azad University, Tehran, Iran; ^6^Department of Medical Emergencies, Qom University of Medical Sciences, Qom, Iran; ^7^Managerial Epidemiology Research Center, Department of Public Health, School of Nursing and Midwifery, Maragheh University of Medical Sciences, Maragheh, Iran; ^8^Department of Community Medicine, Alborz University of Medical Science, Karaj, Iran; ^9^Department of Health Education and Promotion, School of health, Iran University of Medical Sciences, Tehran, Iran; ^10^School of Nursing and Midwifery, Shahroud University of Medical Sciences, Shahroud, Iran

**Keywords:** Physical Inactivity, Systematic Review, Iran

## Abstract

***Introduction:*** Physical inactivity is one of the most important risk factors for chronic diseases, including cardiovascular disease, cancer, and stroke. We aim to conduct a systematic review of the prevalence of physical inactivity in Iran.

***Methods:*** We searched international databases; ISI, PubMed/Medline, Scopus, and national databases Irandoc, Barakat knowledge network system, and Scientific Information Database (SID). We collected data for outcome measures of prevalence of physical inactivity by sex, age, province, and year. Quality assessment and data extraction has been conducted independently by two independent research experts. There were no limitations for time and language.

***Results:*** We analyzed data for prevalence of physical inactivity in Iranian population. According to our search strategy we found 254 records; of them 185 were from international databases and the remaining 69 were obtained from national databases after refining the data, 34 articles that met eligible criteria remained for data extraction. From them respectively; 9, 20, 2 and 3 studies were at national, provincial, regional and local levels. The estimates for inactivity ranged from approximately 30% to almost 70% and had considerable variation between sexes and studied sub-groups.

***Conclusion:*** In Iran, most of studies reported high prevalence of physical inactivity. Our findings reveal a heterogeneity of reported values, often from differences in study design, measurement tools and methods, different target groups and sub-population sampling. These data do not provide the possibility of aggregation of data for a comprehensive inference.

## Introduction

Insufficient physical activity (PA) considered as one of the top 10 leading causes for premature death worldwide.^1^ It is estimated that at least 3.2 million deaths/year are attributable to insufficient PA.^1^ According to the World Health Organization (WHO) estimations, lack of PA contributes to approximately 17% of diabetes and heart disease, 12% of falls accidents in the elderly, and 10% of breast cancers and colon cancers.^1^

Scientific evidence reveals that regular PA is one of the most important preventive factors for chronic diseases, including cardiovascular disease, cancer, and stroke. This role would be more highlighted when we know that these three leading causes of premature death among adults aged 18 years and more.^2-4^

PA defined as any bodily movement that requires energy expenditure it could contains a wide range of working, playing, carrying out household chores, travelling, and engaging in recreational pursuits. It is mentionable that term of “PA” should not be confused with “exercise”, which is a subcategory of PA that is planned, structured, repetitive, and aims to improve or maintain one or more components of physical fitness.^1^

In the past two decades, as a result of some effective health transitional factors, PA has decreased in all of age groups.^5,6^ More than 80% of the world’s adolescent population is insufficiently physically active.^1^ Overweight and obesity in most individuals result from excessive energy consumption as primitive outcomes of physical inactivity.^7^

In this regard, the patterns of related factors are mostly complex, and mostly differ between countries and population sub-groups.^8-10^ On the other hand, for planning, funding, implementation, and management of interventional programs, we must rely on accurate evidence and conducted researches.^11,12^ To monitor, prevent, and control the programs, all of stakeholders call for valid scientific documents.^9^

Based on the best of our knowledge, despite of the priority of problem, there is no comprehensive related literature on these topics.^10,13^ This study aimed to systematically review all of studies on prevalence of physical inactivity in Iranian population. We followed a comprehensive approach to conducting an up-to-date systematic review and meta-analytic comparison of all available studies.

## Methods

### Search strategy

In order to identify prevalence of physical inactivity in Iran, we searched three international databases including, PubMed, ISI/WOS, and Scopus, as well as the national banks of Irandoc, Barakat knowledge network system, and Scientific Information Database (SID) searched for published scientific papers and peer review studies. The search terms for the field of “physical inactivity”, “PA”, “Motor Activity”, “fitness”, “exercise”, “‘energy expenditure” limited national, provincial, district, community population-based studies, human subject, and without restriction on language and time and if there was any non English paper we assessed it through exact translation by experts. There was no limitation on age groups. Based on approved protocol, all of processes followed by two independent research experts and probable discrepancy resolved through referencing the main investigator opinion. The agreement between two independent researchers was reported 80%.

### Inclusion and exclusion criteria

We included all available population based studies that were conducted on physical inactivity. Movement that increases heart rate and breathing or any bodily movement produced by skeletal muscles that result in calorie expenditure considered as definition of PA. Physical Inactivity defined as term used to identify people who do not get recommended sufficient level of regular PA to maintain health.^1^

As we focused on population based studies, studies in sub group specific population such as patients, employees, volunteers, immigrants, and also hospital-based studies excluded. If there were more than one paper that were extracted from one specific study, more complete reported data was considered from only one paper. We also excluded article with duplicate citation and those, based on data refinement results were non relevant.

### Data management

The bibliographic information of searched studies was saved on Endnote software for further reference management. Through three steps of data refinement, including titles, abstracts and full texts review, all of processes follow by two independent experts. Possible disagreements were resolved by discussion and consensus.

### Quality assessment and data extraction

The quality assessment and data extraction of eligible remained papers has been conducted independently by two independent research experts and probable discrepancy between them resolved through referencing the third expert opinion. Quality assessment of general information about the study design, sampling strategy, and measurement quality was assessed through designed form that was approved by scientific committee of project. The final decision was based on the total scores obtained by each paper in ranking scale of: excellent (13-19), good (6-12) or poor (≤ 5). Poor quality papers have been deleted and two other categories considered for data extraction processes. Using Cohen’s kappa statistic, agreement between the results of quality assessment of two experts was 0.92.

Data were collected according to a standard protocol including for citation, place of study, type of study, study year, publication year, sample size, age range, questionnaire or tools, mode of reporting, and sex were extracted based on studied groups.‏

## Results

**Figure 1:**
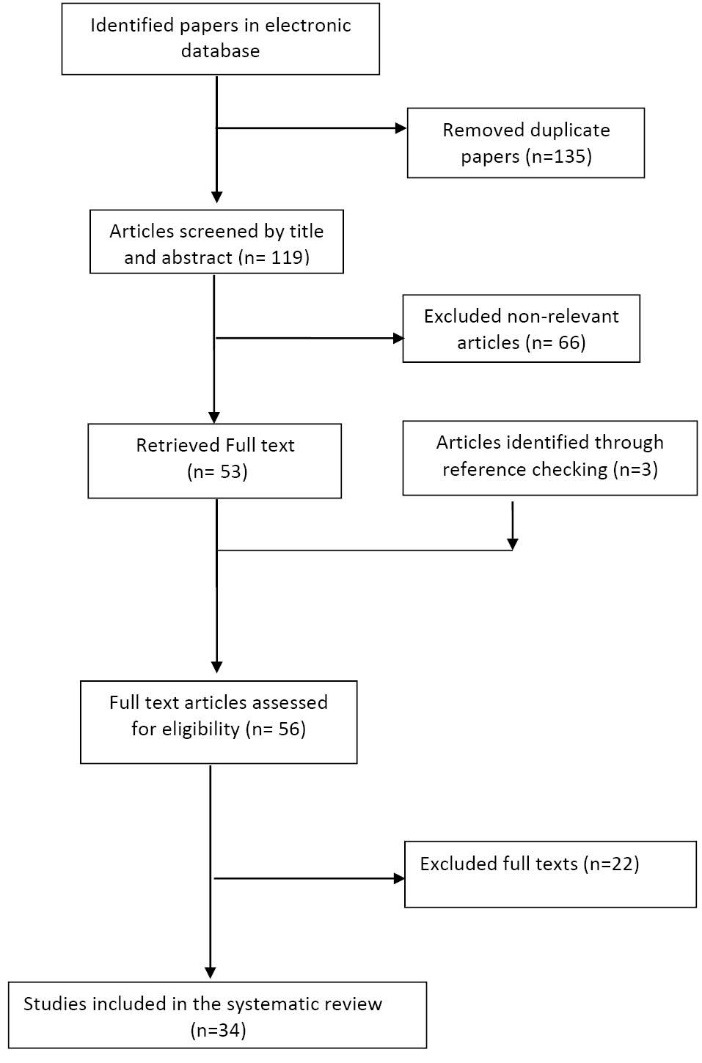
Flowchart for selection of primary study.

We analyzed data for prevalence of physical inactivity in Iranian population. According to our search strategy we found 254 records; of them 185 were from international databases and the remaining 69 were obtained from national databases. After removing duplicates, via the refining steps, only 53 articles were found related to our study domain. Considering inclusion and exclusion criteria, 34 articles that met eligible criteria remained for data extraction. The flow diagram of the study selection process is shown in Figure 1.

All of the studies were cross sectional ones. Tehran Lipid and Glucose Study (TLGS), Isfahan Healthy Heart Program (IHHP) and Persian Gulf Healthy Heart Project (PGHHP) were longitudinal studies which aimed to prevent non-communicable or cardiovascular disease. Golestan Cardiovascular Risk Factors Study (GCRFS) was a cross sectional study aimed to recognize cardiovascular risk factors. All included studies were from primary surveys. Although we included data from Phase II (follow up or interventional) TLGS and IHHP study, all included ones are from baseline data. Also, all studies used interview-administered mode for questionnaire completion.

No single study reported the prevalence of physical inactivity through systematic approach in Iran. Twenty one published studies reported PA throughout Iran. General characteristics of studies are given in Supplementary File 1. All of them were reported or conducted between 1999 and 2012. PA was the primary aim of the study in the 3 studies. PA was reported in a national scale in four studies. Three out of four studies have reported the prevalence of PA in 2005.^14-16^ Another one reported such variable in 2007.^17^

Population based studies varied greatly in reporting PA variables. Three out of five study from IHHP study provided quantitative information in leisure time physical activity (LPA) and/or occupational physical activity (OPA) and/or total PA in terms of the metabolic equivalent of term (MET)-minute/week.^18-20^

Four out of six studies from TLGS data reported percentage of those with light, moderate and heavy PA. The others focused on PA engagement (yes/no; inactive ones).^21,22^ These studies reported different variables of studied behavior. Isfahan reported percentage of physical inactive while North West of Iran provided information on those in different categories of recreational and non-recreational PA. Moreover, Yazd study categorized participants as inactive, sufficient active and highly active.

## Discussion

In our study, from total of 245 searched papers, 34 studies were eligible for inclusion. Our findings provide evidence-based data for better insight for relevant stakeholders. From total 34 included studies, 8 studies were based on individual data and other 26 studies were designed and conducted as national or sub-national surveys. Nine trials were at the national level, 20 studies were at provincial level, 2 surveys at regional level and 3 investigations were assigned to local areas. Six papers did not refer to the year that studies have been run during that. Considering the age ranges of participants; 10 studies covered the age categories of adolescents and youth and other studies focused only on adult target groups. There was a considerable disparity on measurement criteria or tools for physical inactivity. Such a disparity in measurement criteria led to uncooperative results that could not aggregate as deductive evidence gap. Global physical activity questionnaire (GPAQ) and Baecke questionnaire were used as the most popular tools. This is also mentionable that in 16 study results were presented separately for two sexes. The estimations for inactivity with a wide range from near to 30% up to about 70% had considerable variation between sexes and studied sub-groups.

Assessment of health related indicators as well as the estimates of their levels and effects are the most essential requisites for evidence-based health policies.^44^ PA has several physical, psychological and social benefits for all age groups and scientific evidences support of its preventive role for a wide range of physical and mental health problems.^13^

Based on our experience despite of priority of problem and wide range of different related multidisciplinary fields, there is an evident gap in papers and published data. Other relevant studies show different but lower estimation of prevalence of PA. After multivariate analyses estimation of physical inactivity in adult southern Brazilian population, was 41.1%.^45^ Available data from a small number conducted of similar studies suggests a high prevalence of 43.3%–99.5% physical inactivity among Saudi children and adults alike.^46^ This estimation among US adults was 23%, with more women (28%) than men (17%).^47^ There are also some evidences on high estimation in Canada.^48^ The worldwide prevalence of physical inactivity estimated as 21.4% (95% CI: 18.4–24.3), being higher among women (mean= 23.7%, 95% CI: 20.4–27.1) than men (mean= 18.9%, 95% CI: 16.2–21.7).^45,49^ Depending on how PA measure, the results of prevalence are different.^50^

On the other hand, considering the quality of data presentation in published papers it is noticeable that because of quality of reporting, most of them cannot share their finding in whole body of related evidences. Referring to methodological aspects, many papers did not explain about their sampling method, their scope of study, demographic information of target groups, or even time period of the sampling. One of the most other limitation backed to disparities of data based on different measures that PA has been reported by using them. On the other hands many studies focus on sub group population that could not be gathered with representative data.

Shortcomings in accuracy of health measures or even gaps in data presenting methods and skills of designing and conducting the studies, limited our access to targeted accurate reliable data.^51^ Quality of presented data also has an important role in practical use of evidence for estimation and planning of health problems.^52^

Related studies show that physical inactivity is cause of 6%–10% of the major non-communicable diseases of coronary heart disease, type 2 diabetes, and breast and colon cancers.^53,54^ Furthermore, at global level, this causes 9% of premature mortality, and more than 5·3 of the 57 million deaths in 2008.^53^ This is estimated that with elimination of physical inactivity, life expectancy of the world’s population might be expected to increase by 0.68 years.^53,54^

In Iran, estimated population attributable fraction (PAFs), calculated with adjusted relative risks, for coronary heart disease, type 2 diabetes, breast cancer, colon cancer, and all-cause mortality associated with physical inactivity were respectively 6·1% (2.2 to 10.2); 7.6% (3.8 to 11.8); 12·2% (5.8 to 18.9); 10·9% (6.2 to 15.8), and 9·9% (7.9 to 11.9).^53^

Available results emphasize that there is a progressive need for providing the specific health programs, based on targeted specific needs.^12,55,56^

In these regards, physical inactivity extracted from a complex set of physical, psycho-social, cultural known/unknown factors. To designing the effective intervention, we should rely on accurate evidence that are extracted from scientific studies and reliable data.^55,56^

Considering the previous studies, the present study has several achievements. As best of our knowledge, this is the first systematic review on reported prevalence of physical inactivity among the Iranian population according to age and sex. In addition in present study by using comprehensive search terms, all available sources of data and domestic data-bases were searched. As the main limitation, the validity and applicability of our systematic review depends on the quality of conduction and reporting the primary studies that are included. As another considerable point, heterogeneity of searched results limits the generalization of our findings. More over we could not report the prevalence of physical inactivity based on its severity.

Considering above, present finding could provide practical information for better data reporting in papers, and also priorities of researches in this field. We emphasize that planning and evaluation of health strategies must be followed based on reliable data and accurate studies. Aims to that, more reviews on determinants of physical inactivity in different populations are recommended.

## Conclusion

Results of present systematic review showed that prevalence of physical inactivity is high in Iran. Our findings also reveal a heterogeneity in reported values due to diversity in study design, measurement tools, target groups and sub-population sampling. The findings of present study regarding heterogeneity of study design, tools of data collection and study population support future efforts to improve data collection and assessment of PA using a standardized assessment tools such as GPAQ or BRFSS at national and provincial level to provide the possibility of meta-analysis of data for a comprehensive and accurate inference about level of PA in Iran at national and subnational level.

## Ethical approval

Present study was approved by the ethical committee of Tehran University of Medical Science. All of included studies in our review would be cited in all reports and all publications of our study. Whenever we needed more information about a certain study, for obtaining required information, we contacted the corresponding author.

## Competing interests

The authors declare that they have no competing interests.

## Acknowledgments

The authors are thankful of the team working on this study and all participants who made this experience. This project was funding by Alborz University of Medical Sciences.

## Supplementary Files

Supplementary file 1 contains Table S1
