# Supplementary material for: Prevalence of physical inactivity in Iran: a systematic review
Source: J Cardiovasc Thorac Res. 2016 Sep 30;8(3):92–7. doi: 10.15171/jcvtr.2016.20 (PMC5075364; doi:10.15171/jcvtr.2016.20)
Supplement: Supplementary file 1 contains Table S1 [file jcvtr-8-92-s001.pdf]

Table S1. The results of including papers for prevalence of physical inactivity in Iran

No	Citation	Level/ Place of study	Type of study	Year of study	Sample size	Age Range	Diagnosis criteria or tools	Mode of Reporting	Outcome (physical activity)		
									Female	Male	Overall
1	Janghorbani et al(14)	National	Survey	2005	45082 men and 44322 women	15-65	GPAQ	% of subjects with LPA according to never married, married and formerly married	-	-	Never married, 44.9<br>Married, 23.6<br>Formerly married, 16.3<br>Total: 27.93
2	Janghorbani et al(23)	National	Survey	2005	89404	15-65	GPAQ	% of subjects with LPA	20.3	35.4	-
3	Alikhani et al (15)	National	Survey	2005	70,981	25–64	GPAQ	Mean time (95%CI) (minute) spent in OPA per day,<br>Mean time (95%CI) (minute) spent in transport-related activity per day<br>Mean time (95%CI) (minute) spent in recreational PA per day	-	-	27.5 (26.3–28.8)<br>43.8 (42.3–45.4)<br>28.6 (27.3–30.0)

4	Esteghamati et al(24)	National	Survey	2007	3001	25–64	GPAQ	% of subjects with PA	Low: 48.6<br>Moderate: 27.1<br>High: 24.3	Low: 31.6<br>Moderate: 22.3<br>High: 46.1	-
5	Koohpayehzadeh et al(25)	National	Survey	2011	4121	25-64	IPAQ	% (95% CI) of subjects with PA	-	-	Low: 39.1%<br>Moderate: 17.8%<br>High: 43.1%
6	PGHHS 2003, 2004 (26)	Provincial (Bousheher)	Survey	2004	3723	≥ 25	BRFSS	% (95% CI) of subjects with physical inactivity	73.8 (72.34-75.15)	67.0 (65.45-68.49)	-
7	Golestan 2005, 2006	Provincial (*Golesta)n*	Survey (GCRFS)		5000	20-65	running, jogging, swimming, bicycling or walking at least 30 min at a time per day	% (95% CI) of subjects with physical inactivity	79.5 (78.35-80.61)	71.3 (70.02-72.55)	-
8	Sadeghi et al(27)	Provincial (Isfahan)	Individual data		1520	> 15	Minnesota	% (95% CI) of subjects with physical inactivity	32 (29.63-34.38)	22 (19.91-24.14)	-
9	Saidei et al (28)	Provincial (Isfahan)	Survey	2001	4178	-	Baecke	Mean ±SD of LPA (Met-Min. Week), OPA (Met-Min. Week)	460.51±663.32,<br>4498.91±2238.99,	519.46±681.82,<br>3621.68±2667.0	-
10	Sadeghi et al(29)	Provincial	Survey	2002	12514	> 19	Baecke	% (95% CI) of subjects with regular PA	-	-	15.3 (14.66-15.93)

		(Isfahan)									
11	Sarrafzadegan et al(19)	Provincial (Isfahan)	Survey	2000	4178	-	Baecke	% (95% CI) of subjects with moderate or vigorous PA	-	-	50.3 (48.75-51.81)
12	Mousavi et al (30)	Provincial (Isfahan)	Survey	2005	6331 female	-	Baecke	% (95% CI) of Physically inactive subjects	-	-	75.7 (74.61-76.74) [without MS]
13	Bahonar et al(18)	Provincial (Isfahan)	Survey	2001	12541	38.40 ± 14.30	Baecke	% of subjects with LPA	70.3	98.7	-
14	Hajian-Tilaki et al (20)	Regional (North of Iran*)*	Individual	-	1800 women and 1800 men	F:37.5 ±13 and M:38.5±14.3 years old	Not indicated	Exercise (Hours/week) OPA (low, moderate, severe); LPA (very low, low, high, very high) and	≤ 2 h/w: 72%, 3-5≤: 28%/ OPA (low: 59.6%)/ LPA (very low and low:77.2 %)	≤ 2 h/w: 86.4%, 3-5≤: 13.7%/ OPA (low: 69%)/ LPA (very low and low:65.4%)	≤2 h/w: 79.2%, 3- 5≤: 20.8%/ OPA (low: 64.3%)/ LPA (very low and low:81.1%)
15	Hajian-Tilaki et al (31)	Babol	Individual	2008	1200	12-17 14.2±1.7	Baecke	% of subjects with LPA	-	-	The levels of high, moderate and low leisure-time PA were 2.9 %, 79.4 % and 17.7 %, respectively.
16	Dastgiri et al (32)	Regional (North of Iran*)*	Individual	2005	300	> 18	National Health and Nutrition Examination Survey	% (95 % CI) of subjects with recreational PA	Heavy: 7.6 (3.47-12.14) Moderate: 73.8 (66.74-80.28), Light: 18.4 (12.89-25.15)	Heavy:9.8 (5.34-16.25) Moderate: 78 (69.99-84.76), 11.4 (6.50-18.04) Light: 0.8 (0.01-4.14)	-
17	Ghodousi et al(33)	Provincial (Tehran)	Survey (*TLGS, Phase I*)	2001	11000	20-69	Lipid Research Clinic	% (95% CI) of subjects light, moderate, and heavy	-	-	62.8 (61.88-63.70), 13 (12.37-13.64), 24.2 (23.40-25.01)

18	Azadbakht et al (34)	Provincial (Tehran)	Individual	2001	F: 926	40-60	International PA questionnaire	% (95% CI) of subjects with light, moderate, and heavy PA	-	-	Light: 45 (41.68-48.19)<br>Moderate: 30 (26.97-32.97)<br>Heavy: 25 (22.18-27.86)<br><br>n-2007
19	Mirmiran et al(35)	Provincial (Tehran)	Survey	2000	840	18-74	Lipid Research Clinic	% (95% CI) of subjects with light, moderate and heavy PA	-	-	Light: 57 (47.79-64.78), Moderate: 30 (22.55-38.31)<br>Heavy: 13 (7.8-19.55)
20	Mirmiran et al(21)	Provincial (Tehran)	Survey	2000	462	> 16	Lipid Research Clinic	% of subjects with light, moderate and heavy PA	Light: 50<br>Moderate : 31<br>Heavy:19	Light: 55<br>Moderate : 32<br>Heavy:13	-
21	Sabet et al (36)	Provincial (Tehran)	Survey	-	836 male	≥ 20	Lipid Research Clinic	% (95% CI) of subjects with vigorous, moderate PA and inactive	-	Vigorous : 17.8 (15.17-20.46)<br>Moderate : 17.5 (14.94-20.21)<br>Inactive: 64.2 (60.63-67.25)	-

22	Hadaegh et al (22)	Provincial (Tehran)	Survey	-	3444	≥ 20	Lipid Research Clinic	% (95% CI) of subjects with low and high PA	Low: 40.4 (38.74-42.04), High: 59.6 (57.92-61.22)	Low: 37.6 (35.95-39.21), High: 62.4 (62.07-65.31)	-
23	Harati et al(37)	Provincial (Tehran)	Survey (*TLGS, Phase II)*	2005	Control: 5114; Intervention: 3098	> 20	Modifiable Activity Questionnaire	% (95% CI) of subjects with low, moderate and heavy PA	-	-	Low: 66 (64.67-67.29) , Moderate: 12 (11.10-12.90) Heavy: 22 (20.86-23.15)
24	*MONICA*	Provincial (Bousheh r)	Survey	2003	1574	25-64	MONICA	% (95% CI) of subjects with no PA	-	-	16.8 (14.17-17.85)
25	Motefaker et al(38)	Provincial (Yazd)	Individual data	-	1500	> 20	International PA questionnaire	% (95% CI) of subjects with inactivity, sufficient activity and high activity	-	-	67.3 (64.55-69.37), 15 (13.22-16.90), 17.7 (15.13-18.99)
26	The CASPIAN III study	National	Survey	2010	5623 students from 27 provinces	10-18	at least30 minutes duration of exercises per day which was led to heavy sweating or large increase	% of subjects with PA	Low:81.3 High :18.7	Low:88.4 High: 11.6	Low:84.9 High: 15.1

							s in breathing or heart rate				
27	The CASPIAN IV study	National	Survey	2012	14683 from 30 provinces	6-18 years	at least30 minutes duration of exercises per day which was led to heavy sweating or large increases in breathing or heart rate	% of subjects with PA	Inactive: 39.61, Active: 60.39	Inactive: 28.75, Active: 71.25	Inactive: 34.11, Active: 65.89
28	The CASPIAN IV study (the weight disorders determinants study) Age : 6-18 years	National	Survey	2012	22841 from 30 provinces (n girls:11244, n boys: 11597)	6-18 years	at least30 minutes duration of exercises per day which was led to heavy sweating or large increases in breathin	% of subjects with PA	Inactive: 33.42, Active: 66.58	Inactive: 13.84, Active: 86.16	Inactive: 23.48, Active: 76.52

							g or heart rate				
29	Emdadi et al (39)	Local (Hamadan city)	Individual data	2015	866	40-64 years Mean 49.82±6.91	Questionnaire for self-efficacy toward PA	PA (%) (inactive (light level) or not sufficiently active)	Inactive: 57	-	-
30	Moradi et al (40)	Provincial (Kurdistan)	Survey	2005	2494	39.08±14.37	inactivity was defined as a low level of activity, that is, no or low level of PA at home, at work, during leisure, or during outdoor activities and travel	% (95% CI) of Physical inactivity	-	-	16.9 (15, 18)

31	Moradi et al (40)	Provincial (Kurdistan)		2009	997	Mean 39.77±14.24	physically inactivity was defined as a low level of activity, that is, less than 150 minutes of normal PA per week or less than 60 minutes of intense activity per week at home, at work, during leisure, or during outdoor activities and travel	% (95% CI) of physical inactivity	-	-	26.8 (23, 29)
32	Sanaeinasab et al (41)	Local (Khoramabad)	cross-sectional survey	-	1551 (941 boys and 610 girls). 61% boys 39% girls	12-14	e the amount of time devoted to PA during the previous week	% of subjects with physical inactivity	80.9	96.7	87.1

33	Soltanian et al (42)	Provincial (Bousheh r)	Mental Health Study (MHS), a population based study of adolescents	2005	2584 adolescents (1401 male and 1178 female)	15-19	The short-form of the Iranian version of the International PA Questionnaire (IPAQ)	% of subjects with PA	Inaction: 49.4, minimally active:28.3, HEPA-active: 22.3	Inaction: 68.9, minimally active:18.5, HEPA-active: 12.6	Inaction: 58.3, minimally active:23.8, HEPA- active: 17.8
34	Pazoki et al (43)	Provincial (Bousheh r)	individual	-	335 The participants were community members, academic researchers, health care providers, and policy-makers	F: 25–64	To evaluate PA behavior at both registration time and week 8, participants complete a 7-Day PA recall questionnaire based on the BRFSS; USA/CDC, 2002	% of subjects with PA	-	Moderate PA: 26.59 Vigorous PA: 3.61	-

Abbreviation: PA: physical activity ; GPAQ: Global physical activity Questionnaire; IPAQ: International physical activity Questionnaire; CASPIAN: Childhood and Adolescence Surveillance and PreventIon of Adult Non-communicable disease ; BRFSS: Behavioral Risk Factor Surveillance System; PGHHS: Iran Persian Gulf Healthy Heart Study; HEPA: Health-Enhancing PA; MET: Metabolic Equivalent of Task; TLGS: Tehran Lipid and Glucose Study; GCRFS: Golestan Cardiovascular Risk Factors Study; LPA: Leisure Time Physical Activity; OPA: Occupational Physical Activity
